# Furin-mediated cleavage of LRP1 and increase in ICD of LRP1 after cerebral ischemia and after exposure of cultured neurons to NMDA

**DOI:** 10.1038/s41598-019-48279-x

**Published:** 2019-08-13

**Authors:** Mariko Yamada, Hideki Hayashi, Kaori Suzuki, Shoko Sato, Daisuke Inoue, Yui Iwatani, Meiko Ohata, Bo Yuan, Norio Takagi

**Affiliations:** 1 Department of Applied Biochemistry, Tokyo University of Pharmacy and Life Sciences, 1432-1 Horinouchi, Hachioji, Tokyo 192-0392 Japan; 2 Present Address: Laboratory of Pharmacology, School of Pharmacy, Faculty of Pharmacy and Pharmaceutical Sciences, Josai University, 1-1 Keyakidai, Sakado, Saitama 350-0295 Japan

**Keywords:** Cell death in the nervous system, Cellular neuroscience

## Abstract

The *N*-methyl-D-aspartate (NMDA) receptor has been implicated in several neurodegenerative diseases, including stroke. Low-density lipoprotein receptor-related protein 1 (LRP1) plays pivotal roles in endocytosis and signaling in the cell. Immature LRP1 is processed by furin in the trans-Golgi network (TGN) and transported to the cell surface as its mature form. Activation of mature LRP1 exerts a protective effect against glutamate-induced degeneration of the rat retinal ganglion cells, as was shown in our previous study. However, the roles of LRP1 in the pathogenesis of excitotoxic neuronal injuries remain to be determined. The aim of this present study was to achieve further insight into the pathophysiologic roles of LRP1 after excitotoxic neuronal injuries. Our findings are the first to demonstrate that LRP1 was significantly cleaved by furin after cerebral ischemia in rats as well as after exposure of cultured cortical neurons to NMDA. It was noteworthy that the intracellular domain (ICD) of LRP1 was co-localized with TGN and furin. Furthermore, a furin inhibitor inhibited the cleavage of LRP1 and co-localization of LRP1-ICD with TGN or furin. Our findings suggest that furin-mediated cleavage of LRP1 and changes in the localization of LRP1-ICD were involved in the excitotoxic neuronal injury.

## Introduction

Ischemic brain damage is associated with various deleterious events such as glutamate excitotoxicity, oxidative stress and trophic factor deficiency. An excessive amount of extracellular glutamate induced by cerebral ischemia is a major factor that induces intracellular Ca^2+^ overload via the *N*-methyl-D-aspartate (NMDA) receptor, which is a glutamate receptor, and then causes neuronal cell death. Thus the increase in intracellular Ca^2+^ through the NMDA receptor has been implicated in acute and chronic neurodegenerative diseases, including ischemic stroke, Parkinson’s disease, Alzheimer’s disease, and amyotrophic lateral sclerosis^1–6^. Therefore, it is an important objective to develop therapeutic strategies to prevent NMDA receptor-mediated excitotoxicity and to determine its underlying mechanism.

Low-density lipoprotein receptor-related protein 1 (LRP1) is highly expressed in the central nervous system^7–9^ and functions as not only an endocytic receptor^10,11^, but also a signaling receptor on the cell membrane^12^. In addition, activation of LRP1 by lipoproteins (LPs) exerts a protective effect against glutamate-induced degeneration of the rat retinal ganglion cells (RGCs) in primary cultures and in animal models of glaucoma, as was reported in our previous study^13^. Therefore, LRP1 is suggested to be a key role in the pathogenesis of excitotoxic cell injuries.

Immature LRP1 is processed by furin in the trans-Golgi network (TGN) and transported to the cell surface as its mature form consisting of α-chain and β-chain. Mature LRP1 is further processed by other enzymes, for example, γ-secretase, matrix metalloproteinase, and PCSK9^14^. Then, the intracellular domain (ICD) is produced^15^. It has been shown that cleaved ICD is transported into the nucleus where it contributes to transcriptional regulation of target genes. These findings raise the possibility that LRP1 and/or LRP1-ICD may contribute to the pathogenesis in several types of diseases. In this sense, we interestingly demonstrated earlier that furin participates in NMDA-induced neuronal death by acting upstream of calpain^16^. Calpain is known to be involved in Ca^2+^-dependent neurodegeneration, and inhibition of calpain can lead to neuroprotection *in vitro*. Therefore, pathophysiological changes in LRP1 and furin may possibly be associated with ischemic brain injury.

The aim of this study was to obtain further insight into the pathophysiologic roles of LRP1. We conducted experiments to determine changes in the processing pathway of LRP1 in a rat model of cerebral ischemia and by examining cultured cortical neurons after NMDA receptor-mediated excitotoxicity, which has been implicated in a variety of neurodegenerative diseases.

## Results

**Figure 1:**
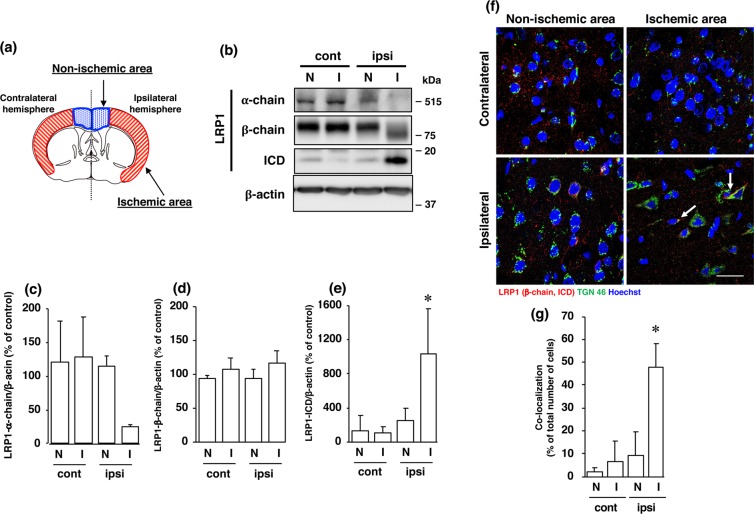
(**a**) Schematic drawing of brain regions used in the experiments. (**b**) Changes in α-chain, β-chain, and ICD of LRP1 at 24 h after MCAO/R. Proteins from non-ischemic (N) and ischemic (I) areas from the ipsilateral (ipsi) and contralateral (cont) cortex in MCAO/R rats were analyzed by Western blotting with anti-α-chain and anti-β-chain/ICD of LRP1, and anti-β-actin antibodies. Cropped blots are displayed and full-length blots are presented in Supplementary Fig. S1. Bands corresponding to α-chain (**c**), β-chain (**d**), and ICD (**e**) of LRP1 and β-actin were scanned, and the scanned bands were normalized by the untreated naïve control on the same blot. β-Actin was used as a loading control. Results are the means ± SD (n = 3 rats per group). *Indicates a significant difference from the corresponding area of the contralateral hemisphere (p < 0.05). (**f**) Changes in localization of β-chain and/or ICD of LRP1 and TGN at 24 h after MCAO/R. Non-ischemic and ischemic areas of the ipsilateral and contralateral hemispheres in the brain of MCAO/R rats were immunostained with anti-TGN46 and anti-β-chain/ICD of LRP1 antibodies, and then observed with a confocal microscope. The colocalization of TGN46 with LRP1-ICD is indicated by the “white arrow”. Representative images are shown from one rat. The scale bar represents 30 µm. (**g**) Changes in localization of β-chain and/or ICD of LRP1 and TGN at 24 h after MCAO/R. The number of LRP1-ICD immune-positive cells that co-localized with TGN46 was counted. Results are expressed as the means ± SD (n = 4 rats per group). *Indicates a significant difference from the corresponding area of the contralateral hemisphere (P < 0.05). The range of the number of cells counted for N-cont, I-cont, N-ipsi, and I-ipsi were 22–60/rat (the total number of cells counted: 150), 14–66/rat (total: 139), 22–59/rat (total: 159), and 23–63/rat (total: 156), respectively. The total number of cells counted in Fig. 1g was 604.

As the LRP1 precursor (~600 kDa) is cleaved by furin in the TGN to generate a 515-kDa α chain (LRP1-α-chain) and an 85-kDa membrane-anchored cytoplasmic β chain (LRP1-β-chain), we determined the amounts of α-chain and β-chain of LRP1 proteins after cerebral ischemia. The amount of α-chain of LRP1 was decreased (Fig. 1b,c), whereas that of a 17-kDa ICD, which is produced by cleavage at an intramembrane site of LRP1, was significantly increased in the ischemic areas of the ipsilateral hemisphere after cerebral ischemia (Fig. 1b,e). The level of β-chain of LRP1 was not altered after cerebral ischemia, although its molecular weight was shifted toward a lower one in the ischemic areas (Fig. 1b,d). Furthermore, immunohistochemical analysis showed that LRP1-ICD, which could be recognized by anti-LRP1 C-terminal antibody, was merged with TGN marker TGN46, in some cells in the ischemic area, but not in the non-ischemic area of the ipsilateral hemisphere (Fig. 1f, Supplementary Fig. S7). The number of cells that LRP1-ICD was co-localized with TGN marker TGN46 was significantly increased in the ischemic areas of the ipsilateral hemisphere after cerebral ischemia (Fig. 1g).

**Figure 2:**
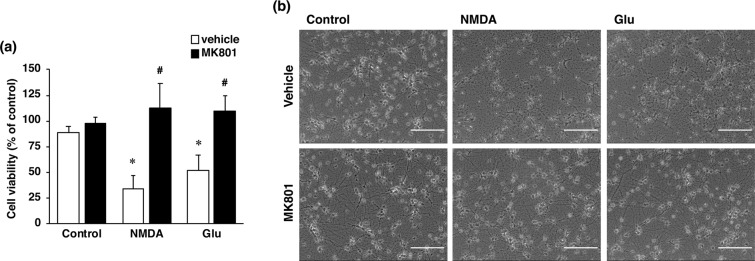
(**a**) Effect of NMDA receptor antagonist on NMDA- and glutamate (Glu)-induced cell injury. Cell viability in cultures of 30 µM NMDA- and 300 µM glutamate-treated cells without (white bars) or with (black bars) 10 µM MK801. The relative cell viability was expressed as the percentage of the absorbance at 450 nm of each treatment group against that of the untreated control group. Results are the means ± SD (n = 3 independent experiments). *Indicates a significant difference from the untreated group (p < 0.05); and #, a significant difference from the NMDA-treated and MK801-untreated group or glutamate-treated and MK801-untreated group (p < 0.05). (**b**) Effect of NMDA receptor antagonist on NMDA- and glutamate-induced cell injury. Representative images are shown from one experiment. The scale bar represents 100 µm.

**Figure 3:**
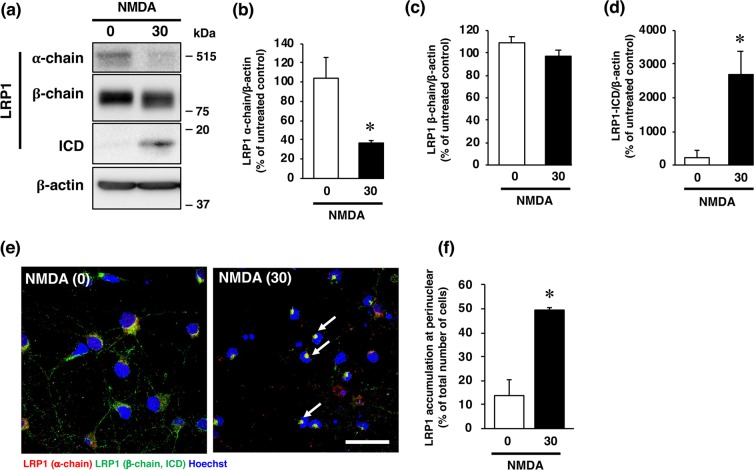
Effects of NMDA treatment on protein levels of α-chain, β-chain, and ICD of LRP1 at 4 h after 0 µM (0) or 30 µM (30) NMDA treatment. Proteins from cortical neurons in primary culture were analyzed by Western blotting with anti-α-chain and anti-β-chain/ICD of LRP1, and anti-β-actin antibodies (**a**). Cropped blots are displayed and full-length blots are presented in Supplementary Fig. S2. Bands corresponding to the α-chain (**b**), β-chain (**c**), and ICD (**d**) of LRP1 and β-actin were scanned, and the scanned bands were normalized by reference to the untreated control on the same blot. β-Actin was used as a loading control. Results are the means ± SD (n = 3 independent experiments). *Indicates a significant difference from the NMDA-untreated group (p < 0.05). (**e**) Effects of NMDA treatment on localization of α-chain and β-chain/ICD at 4 h after 0 µM (0) or 30 µM NMDA (30) treatment. Cortical neurons in primary culture were treated with NMDA were immunostained with anti-α-chain (red) and anti-β-chain/ICD (green), which recognizes ICD domain, of LRP1 antibodies. Representative images are shown from one experiment. The scale bar represents 30 µm. (**f**) The number of cells where LRP1-ICD was localized in the perinuclear region was counted. Results are expressed as the means ± SD of 4 independent experiments. *Significant difference from the NMDA-untreated group (NMDA [0]) (P < 0.05). The range of the number of cells counted for NMDA [0] and NMDA [30] were 28–42/experiment (the total number of cells counted: 138) and 32–87/experiment (total: 144), respectively. The total number of cells counted in Fig. 3f was 282.

**Figure 4:**
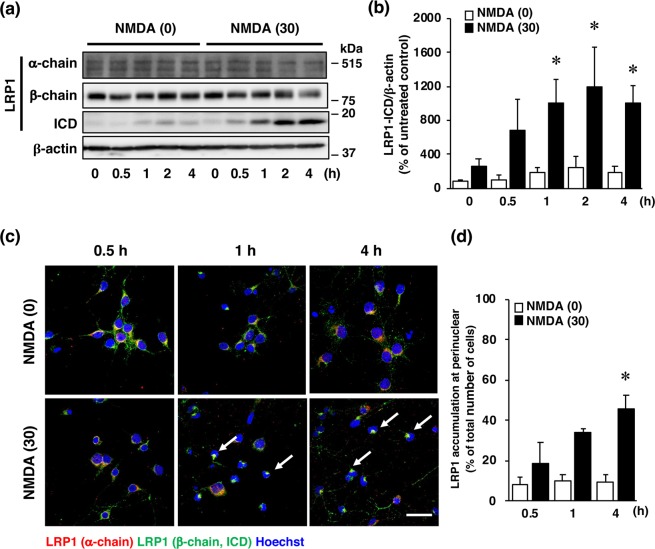
(**a**) Effects of NMDA treatment on the levels of α-chain, β-chain, and ICD of LRP1 at 0, 0.5, 1, 2, and 4 h after 0 µM (0) or 30 µM (30) NMDA treatment. Proteins from cortical neurons in primary culture were analyzed by Western blotting with anti-α-chain and anti-β-chain of LRP1, and anti-β-actin antibodies. Cropped blots are displayed and full-length blots are presented in Supplementary Fig. S3. (**b**) Bands corresponding to ICD of LRP1 and β-actin were scanned, and the scanned bands were normalized by reference to the untreated control on the same blot. β-Actin was used as a loading control. Results are the means ± SD (n = 3 independent experiments). *Indicates a significant difference from the NMDA-treated group at 0 h (p < 0.05). (**c**) Effects of NMDA treatment on localization of α-chain and β-chain/ICD of LRP1 at 0.5, 1, and 4 h after 0 µM (NMDA [0]) or 30 µM NMDA (NMDA [30])) treatment. Representative images are shown from one experiment. The scale bar represents 30 µm. (**d**) The number of cells where LRP1-ICD was localized in the perinuclear region was counted. Results are expressed as the means ± SD of 4 independent experiments. *Significant difference from the NMDA-treated group at 0.5 h (NMDA [30]) (P < 0.05). The range of the number of cells counted in NMDA [0]-0.5 h, NMDA [0]-1 h, NMDA [0]-4 h, NMDA [30]-0.5 h, NMDA [30]-1 h, and NMDA [30]-4 h, were 20–32/experiment (the total number of cells counted: 105), 30–57/experiment (total: 172), 18–35/experiment (total: 101), 27–74/experiment (total: 195), 19–40/experiment (total: 113), and 31–57/experiment (total: 174), respectively. The total number of cells counted in Fig. 4d was 860.

**Figure 5:**
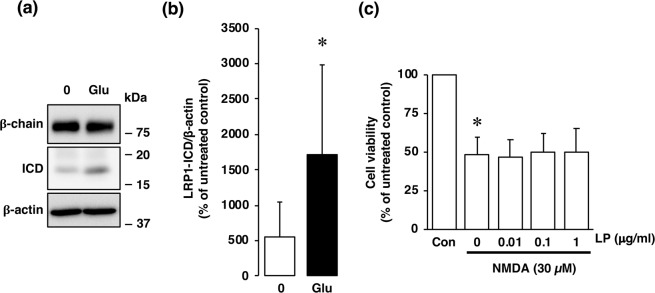
(**a**) Effects of glutamate treatment on protein levels of β-chain and ICD of LRP1 at 4 h after 0 µM (0) or 30 µM (Glu) glutamate treatment of cortical neurons in primary cultures. Proteins from the cultures were analyzed by Western blotting with anti-β-chain/ICD of LRP1 and anti-β-actin antibodies. Representative blots of β-chain and ICD of LRP1 are shown (**a**). Cropped blots are displayed and full-length blots are presented in Supplementary Fig. S4. Bands corresponding to ICD of LRP1 and β-actin were scanned, and the scanned bands were normalized by referring to the untreated control on the same blot (**b**). β-Acitn was used as a loading control. Results are the means ± SD (Cortical neuron n = 8 independent experiments). *Indicates a significant difference between 0 µM and 30 µM glutamate treatments (p < 0.05). (**c**) Effect of NMDA treatment on cell viability of cortical neurons. Cortical neurons were incubated with Glia-derived ApoE-containing lipoproteins (LP) and/or 30 µM NMDA for 15 min. The relative cell viability was expressed as the percentage of the absorbance at 450 nm of each treatment group against that of the untreated control group. Results are the means ± SD (n = 4 independent experiments). *Indicates a significant difference from the NMDA- and LP-untreated control group (Con; p < 0.05).

It has been well recognized that an excessive influx of Ca^2+^ through the NMDA receptor is related to pathogenesis of diverse neurological and neurodegenerative disorders, including stroke. In agreement with the results of our previous study^16^, the viability of cultured cortical neurons was decreased by exposure of the cells to a concentration of 30 µM NMDA or 10 µM glutamate (Fig. 2a,b); and this decreased cell viability was inhibited by the non-competitive NMDA receptor antagonist MK-801 (Fig. 2a,b). We next examined whether changes in the level of LRP1 protein occurred in neurons with over-activated NMDA receptors. Treatment with NMDA markedly decreased the amount of the α-chain of LRP1, which is the extracellular ligand-binding domain (Fig. 3a,b); whereas the amount of the β-chain of LRP1 was unaffected by the treatment (Fig. 3a,c). The level of the ICD of LRP1 was significantly increased after NMDA treatment (Fig. 3a,d). By immunocytochemical analysis, LRP1 was shown to be widely distributed in the cytosol and dendrites in non-treated neurons (Fig. 3e). After NMDA exposure, LRP1, which was localized at the dendritic spine-like structures, had largely disappeared (Fig. 3e). Interestingly, ICD of LRP1 was localized in the perinuclear region after NMDA treatment (Fig. 3e, Supplementary Fig. S8). The number of cells that ICD was localized in the perinuclear region was significantly increased after NMDA exposure (Fig. 3f). The amount of the ICD of LRP1 started to increase at 1 h and remained significantly higher than the control (0 h) for up to 4 h after NMDA treatment (Fig. 4a,b), and the accumulation of LRP1-ICD in the perinuclear region was evident from 4 h after NMDA treatment (Fig. 4c,d). Treatment with glutamate also increased the amount of the ICD of LRP1 in cortical neurons (Fig. 5a,b). In previous studies, it was demonstrated that treatment with LPs prevented cell injury induced by glutamate treatment in RGCs via intracellular signaling involving LRP1^13,17^. Therefore, we next examined the effects of LPs on excitotoxic injury of cortical neurons. Treatment with LPs did not prevent cell injury in cortical neurons (Fig. 5c).

**Figure 6:**
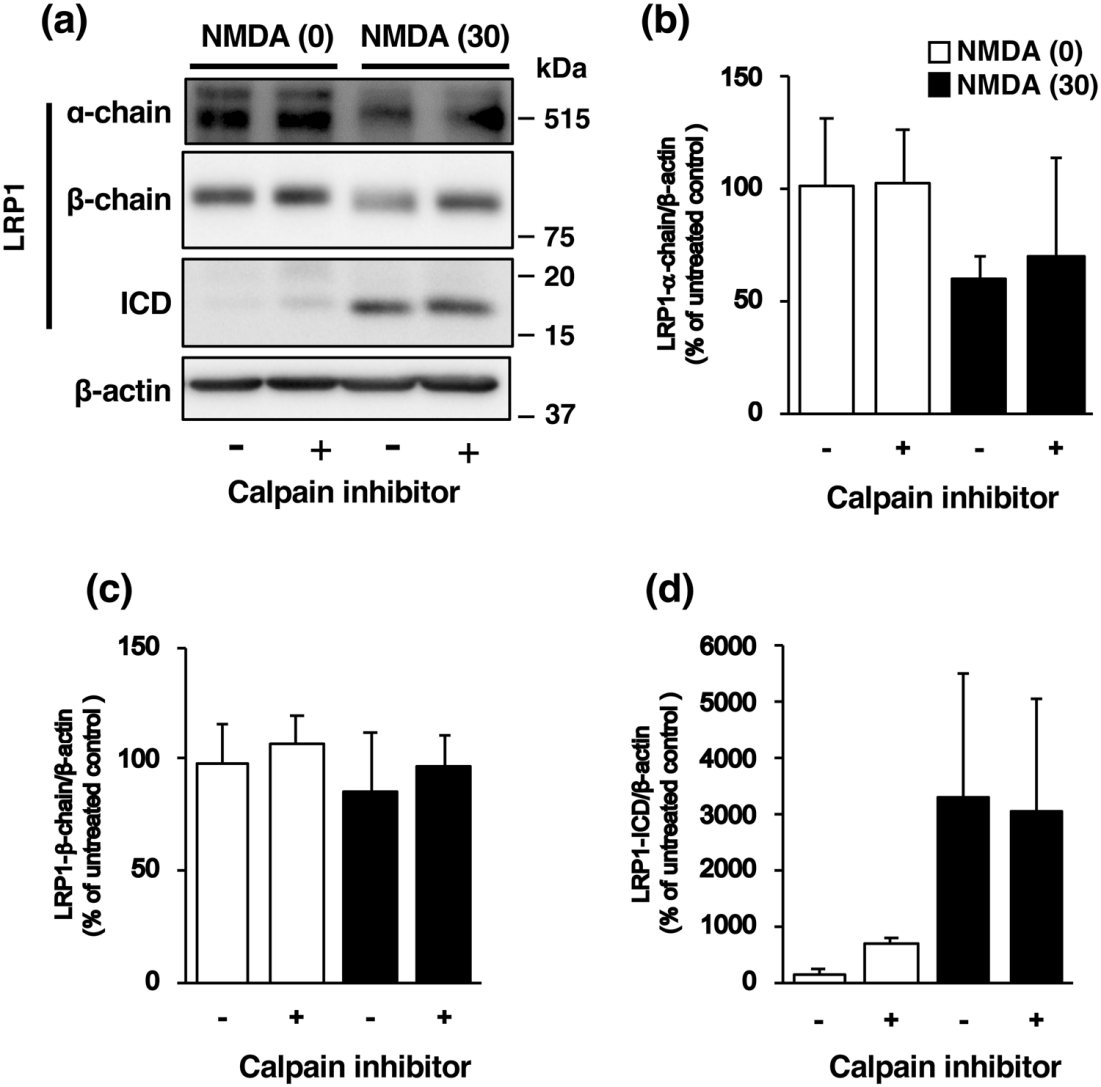
(**a**) Effects of calpain inhibitor (calpeptin) on the levels of α-chain, β-chain and ICD of LRP1 at 4 h after 0 µM (0) or 30 µM (30) NMDA treatment with (+) or without (−) 30 µM calpain inhibitor. Proteins from cortical neurons in primary culture were analyzed by Western blotting with anti-α-chain and anti-β-chain/ICD of LRP1, and anti-β-actin antibodies. Cropped blots are displayed and full-length blots are presented in Supplementary Fig. S5. Bands corresponding to α-chain (**b**), β-chain (**c**), and ICD (**d**) of LRP1 and β-actin were scanned, and the scanned bands were normalized by reference to the untreated control on the same blot. β-Actin was used as a loading control. Results are the means ± SD (n = 4 independent experiments).

**Figure 7:**
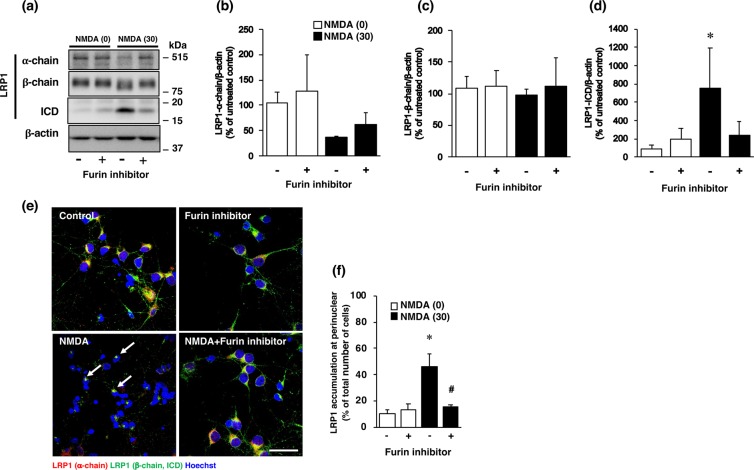
(**a**) Effects of furin inhibitor on the levels of α-chain, β-chain, and ICD of LRP1 at 4 h after 0 µM (0) or 30 µM (30) NMDA treatment with (+) or without (−) 10 µM furin inhibitor. Proteins from cortical neurons in primary culture were analyzed by Western blotting with anti-α-chain and anti-β-chain of LRP1/ICD, and anti-β-actin antibodies. Cropped blots are displayed and full-length blots are presented in Supplementary Fig. S6. Bands corresponding to α-chain (**b**), β-chain (**c**), and ICD (**d**) of LRP1 and β-actin were scanned, after which the scanned bands were normalized by referring to the untreated control on the same blot. β-Actin was used as a loading control. Results are the means ± SD (n = 3 independent experiments). *Indicates a significant difference between 0 µM and 30 µM NMDA treatments without furin inhibitor (p < 0.05). (**e**) Effect of furin inhibitor on localization of α-chain (red) and β-chain of LRP1/ICD (green) at 4 h after 0 µM (control) or 30 µM NMDA (NMDA) treatment with or without 10 µM furin inhibitor. Representative images are shown from one experiment. The scale bar represents 30 µm. (**f**) The number of cells where LRP1-ICD was localized in the perinuclear region was counted. Results are expressed as the means ± SD of 4 independent experiments. *Significant difference from the NMDA- and furin inhibitor-untreated group (NMDA [0] and furin inhibitor [−]) (P < 0.05). ^#^Significant difference from the NMDA-treated and furin inhibitor-untreated group (NMDA [30] and furin inhibitor [−]) (P < 0.05). The range of the number of counted cells in NMDA [0]-furin inhibitor [−], NMDA [0]-furin inhibitor [+], NMDA [30]-furin inhibitor [−], and NMDA [30]-furin inhibitor [+], were 14–36/experiment (the total number of cells counted: 110), 24–38/experiment (total: 128), 38–73/experiment (total: 216), and 27–57/experiment (total: 151), respectively. The total number of cells counted in Fig. 7f was 605.

**Figure 8:**
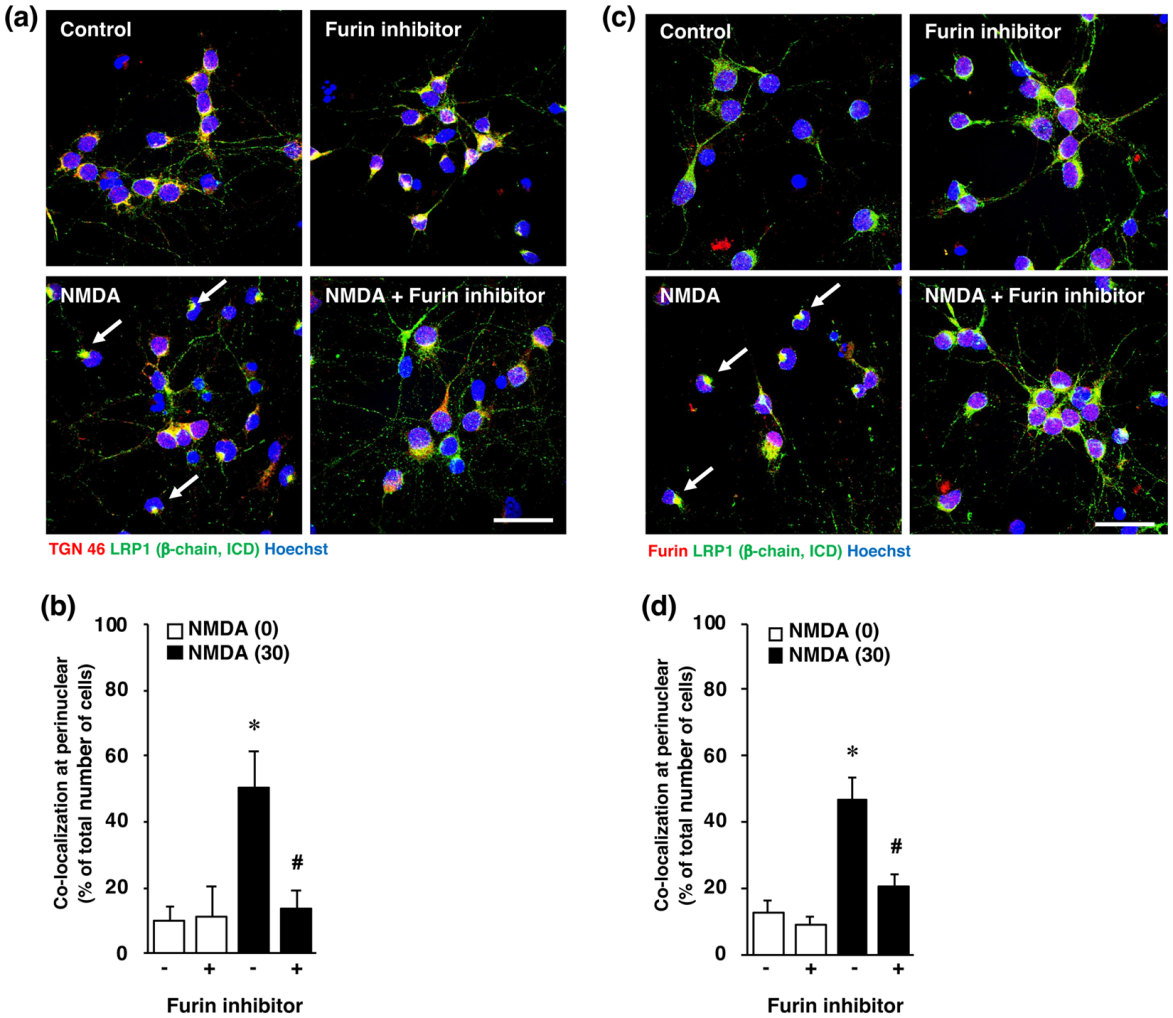
(**a**) Effect of furin inhibitor on localization of TGN46 and LRP1-ICD at 4 h after 0 µM (control) or 30 µM NMDA (NMDA) treatment of cortical neurons with or without 10 µM furin inhibitor. The cells were immunostained with anti-TGN46 and anti-β-chain/ICD antibodies. Representative images are shown from one experiment. The scale bar represents 30 µm. (**b**) The number of LRP1-ICD immune-positive cells that co-localized with TGN46 in the perinuclear region was counted. Results are expressed as the means ± SD of 4 independent experiments. *Significant difference from the NMDA- and furin inhibitor-untreated group (NMDA [0] and furin inhibitor [−]) (P < 0.05). ^#^Significant difference from the NMDA-treated and furin inhibitor-untreated group (NMDA [30] and furin inhibitor [−]) (P < 0.05). The range of the number of counted cells in NMDA [0]-furin inhibitor [−], NMDA [0]-furin inhibitor [+], NMDA [30]-furin inhibitor [−], and NMDA [30]-furin inhibitor [+], were 29–40/experiment (the total number of cells counted: 133), 22–27/experiment (total: 98), 51–82/experiment (total: 186), and 20–32/experiment (total: 109), respectively. The total number of cells counted in Fig. 8b was 526. (**c**) Effect of furin inhibitor on localization of furin and LRP1-ICD at 4 h after 0 µM (control) or 30 µM NMDA (NMDA) treatment with or without 10 µM furin inhibitor. The cells were immunostained with anti-furin and anti-β-chain of LRP1/ICD antibodies. Representative images are shown from one experiment. The scale bar represents 30 µm. (**d**) The number of LRP1-ICD immune-positive cells that co-localized with furin in the perinuclear region was counted. Results are expressed as the means ± SD of 4 independent experiments. *Significant difference from the NMDA- and furin-untreated group (NMDA [0] and furin inhibitor [−]) (P < 0.05). ^#^Significant difference from the NMDA-treated and furin inhibitor-untreated group (NMDA [30] and furin inhibitor [−]) (P < 0.05). The range of the number of counted cells in NMDA [0]-furin inhibitor [−], NMDA [0]-furin inhibitor [+], NMDA [30]-furin inhibitor [−], and NMDA [30]-furin inhibitor [+], were 17–27/experiment (the total number of cells counted: 91), 17–33/experiment (total: 110), 27–63/experiment (total: 179), and 19–40/experiment (total: 111), respectively. The total number of cells counted in Fig. 8d was 491.

It is known that NMDA-induced neuronal injury is caused by excessive calcium influx and that the calcium-activated protease calpain plays a pivotal role in NMDA-induced neurotoxicity. Therefore, to determine the mechanisms underlying the expression of LRP1-ICD, we examined the effect of calpeptin, a calpain inhibitor, on the expression of LRP1-ICD after exposure of cortical neurons to NMDA. Treatment with calpeptin did not affect NMDA-induced expression of LRP1-ICD (Fig. 6a,d), nor the amounts of α-chain (Fig. 6a,b) and β-chain (Fig. 6a,c) of LRP1, in cortical neurons. We previously demonstrated that furin is involved in NMDA-induced neuronal injury by acting upstream of calpain^16^. Next we examined the effects of a furin inhibitor on the amount of LRP1-ICD after NMDA exposure. The increase in the level of LRP1-ICD induced by NMDA exposure was inhibited by treatment with the furin inhibitor (Fig. 7a,d), although there was no statistically significant difference that might have been due to large variations in the level of NMDA-induced ICD expression. The levels of α-chain and β-chain of LRP1 were not affected by treatment with the furin inhibitor (Fig. 7a–c). We furthermore demonstrated that the perinuclear localization of LRP1 C-terminal after NMDA exposure was attenuated by treatment with the inhibitor (Fig. 7e,f). It has been shown that furin trafficks to the TGN or is recycled to the cell surface through the endosomal system^18^. Immunocytochemical analysis showed that the LRP1-ICD was co-localized with TGN marker TGN46 in the perinuclear region after NMDA exposure (Fig. 8a, Supplementary Fig. S9). The number of cells that LRP1-ICD was co-localized with TGN marker TGN46 was significantly increased after NMDA exposure (Fig. 8b). The LRP1-ICD was also co-localized with furin in the perinuclear region (Fig. 8c, Supplementary Fig. S10) and the number of cells that LRP1-ICD was merged with furin was also significantly increased after NMDA exposure (Fig. 8d). We further examined whether the furin inhibitor would affect the localization of the LRP1 C-terminal, which involves LRP1-ICD after NMDA-induced excitotoxicity. The results demonstrated that the furin inhibitor attenuated this co-localization of the LRP1-ICD with the TGN marker TGN46 (Fig. 8a,b) and also lessened that of it with furin after NMDA-induced excitotoxicity (Fig. 8c,d).

## Discussion

It has been shown that LRP plays important roles in the pathogenesis of Alzheimer’s disease^19^. In addition, γ-secretase-mediated proteolysis of LRP1 has been implicated in cerebral ischemia-induced cell death^20^. Although these findings imply that LRP1 may contribute to the development of diseases of the central nervous system, pathophysiological alterations in LRP1 remain to be determined as regarding ischemic brain injury. In the present study, we sought to determine the effect of cerebral ischemia *in vivo* on the level of LRP1 protein. Our results demonstrate for the first time that the expression of LRP1-ICD was significantly increased in the ischemic areas of the ipsilateral cortex, whereas that of the α-chain of LRP1 was markedly decreased comparing to their levels in the contralateral cortex. Furthermore, LRP1-ICD could be co-localized with TGN marker TGN46 in the ischemic regions, but not in the non-ischemic region, of the ipsilateral cortex. These findings raise the possibility that changes in the intracellular signaling via LRP1 and/or the expression of LRP1-ICD play a key role in the pathology of cerebral ischemia.

Thus, by using cortical neurons in primary culture we next investigated the mechanisms by which the expression of LRP1-ICD was increased after NMDA-induced neurotoxicity, which has been implicated in a variety of neurodegenerative diseases. Initially we confirmed that NMDA-induced neuronal injury was mediated through the NMDA receptor^16^. Here we also demonstrated for the first time in cultured cortical neurons that the levels of α-chain of LRP1 in cultured cortical neurons was decreased but that of LRP1-ICD was significantly increased, after NMDA-induced neurotoxicity. As NMDA-induced neurotoxicity is mediated by excessive calcium influx, we examined the effects of calpeptin, an inhibitor of calcium-dependent calpain activation, on the expression of LRP1-ICD, to determine the mechanisms of the increased expression of LRP1-ICD. Treatment with calpeptin did not affect the expression of LRP1-ICD after NMDA exposure, indicating that the increase in the expression of LRP1-ICD by NMDA-induced neurotoxicity was likely to have been caused by factors other than calcium-activated protease calpain. LRP1 is synthesized as a proprotein that undergoes post-translational proteolytic processing, which occurs in the trans-Golgi compartment and is catalyzed by the endopeptidase furin^21^. This processing results in the formation of mature LRP1, which is a noncovalently associated heterodimer consisting of the extracellular α-chain and the transmembrane β-chain, the latter of which includes the ICD sequence. We previously demonstrated that a furin inhibitor protected cortical neurons against NMDA-induced neuronal cell death^16^. Furin is localized in the TGN and contributes to the processing of proNGF and proBDNF, leading to their maturation^22–24^. Furthermore, as furin is necessary for proteolytic processing of LRP1^21^, we next determined the effect of a furin inhibitor on the levels of α-chain of LRP1 and LRP1-ICD in cortical neurons after NMDA exposure. We found it noteworthy that the furin inhibitor attenuated the decrease in the level of the α-chain of LRP1 and the expression of LRP1-ICD after NMDA exposure.

It has been reported that LRP1-ICD is translocated to the nucleus by treatment with LPS^15^. This translocated LRP1-ICD interacts with interferon regulatory factor 3 (IRF-3) to enhance the nuclear export and proteasomal degradation of IRF-3 was enhanced^15^. Furthermore, LRP1-ICD is a part of the transcriptional complex that assembles on the promoter of *ifn-*γ, through which it represses LPS-induced gene transcription *in vivo*^15^. In this sense, it has been suggested that interferon γ is involved in the excitotoxic injury^25,26^. Therefore, although we did not detect LRP1-ICD in the nuclei after NMDA exposure, our finding raises the possibility that the increase in the expression of LRP1-ICD after NMDA exposure might be, in part, associated with IFN-induced neuronal cell injury. Interestingly, in the RGCs, LRP1-ICD was not detected after excitotoxic injury in the previous study^13^. Therefore, our results may provide insights into the differential roles of LRP1 in RGCs and cortical neurons under pathophysiological conditions. In this regard, the reduction in the level of α-chain of LRP1 and the significant increase in that of LRP1-ICD induced by NMDA exposure might be insufficient to mediate intracellular signaling for LP-mediated protection against NMDA-induced neurotoxicity in cortical neurons. Because the α-chain of LRP1 contains the ligand-binding domain, not only internalization of bound ligands but regulation of intracellular signaling through LRP1 could be impaired in NMDA-induced neurotoxicity. Binding of tissue plasminogen activator or α-2-macroglobulin to LRP1 activates Akt and ERK1/2 by a pathway that requires Src family kinases through transactivation of Trk receptors^27^, implying that this LRP1 signaling in neurons might be involved in repair of injuries in the nervous system^27^. Therefore, an impairment of intracellular signaling mediated by LRP1 cleavage might be correlated with our results, indicating that LPs do not have protective effects against NMDA-induced neurotoxicity in cortical neurons. It was recently reported that disruption of Golgi-to-plasma membrane trafficking induces Golgi membrane-associated degradation, by which undelivered cargo molecules are degraded in lysosomes^28^. In the present study, LRP1 was co-localized with the TGN marker TGN46 and furin in the perinuclear region after NMDA exposure. Therefore, it is possible that Golgi membrane-associated degradation occurs in NMDA-induced neurotoxicity that might be associated with furin-mediated degradation of LRP1 in the TGN. Although our data suggested that LRP1 was cleaved by furin after NMDA exposure, it will be necessary to determine the relationship between inhibition of LRP1 cleavage and protective effects of the furin inhibitor per se against NMDA exposure.

In conclusion, our findings are the first to demonstrate that mature LRP1 is cleaved by furin after cerebral ischemia in rats and following NMDA exposure in cortical neurons in primary culture. We considered it noteworthy that the level of LRP1-ICD, which was increased *in vitro* and under ischemic conditions *in vivo*, co-localized with TGN and furin. Furthermore, the cleavage of LRP1 and co-localization of LRP1-ICD with TGN and/or furin were attenuated by the furin inhibitor, which might be associated with protection of cortical neurons against ischemic injury. For the development of therapeutic drugs against ischemic brain injury, additional investigation will be needed to understand the role of LRP1 signaling, including the function of LRP1-ICD, in the normal nervous system and in pathological conditions both *in vitro* and *in vivo*.

## Material and Methods

### Materials

Furin inhibitor 1 (Cat. No. 14965) was purchased from Cayman Chemical (Ann Arbor, MI, USA). MK-801 (Cat. No. M107) was obtained from Sigma Aldrich (St. Louis, MO, USA). Calpeptin (Cat. No. 03-34-0051) was procured from Millipore (Billerica, MA, USA). Other cell culture reagents and medium were from Invitrogen (Carlsbad, CA, USA).

### Animals

Female Sprague-Dawley (SD) rats (embryonic day 16; SLC, Shizuoka, Japan) for preparing primary cultures of rat cortical neurons and male SD rats (7 weeks old, weighing between 200 and 220 g; SLC, Shizuoka, Japan) for an animal model of cerebral ischemia were used in the present study. The rats were maintained at 23 ± 1 °C in a room with a constant humidity of 55 ± 5% and a cycle of 12 h of light and 12 h of dark, and had free access to food and water according to the National Institute of Health Guide for the Care and Use of Laboratory Animals and the Guideline for Experimental Animal Care issued by the Prime Minister’s Office of Japan. All experimental procedures were approved by the Committee of Animal Care and Welfare of Tokyo University of Pharmacy and Life Sciences.

### Animal surgical procedures

Transient focal ischemia was induced by the method described previously^29^ with minor modifications. Briefly, anesthesia was induced with 5% isoflurane and maintained with 2.5% isoflurane. The surgical area was exposed, and then a 4–0 nylon surgical suture with a silicon-coated tip was inserted from the right external carotid artery to the origin of the right middle cerebral artery for occlusion. After 90 min of occlusion, the suture was removed to permit reperfusion. The behavior of the rats was evaluated according to the method of Bederson *et al*.^30^. The rats demonstrating consistent circling toward the contralateral side and a reduced resistance to a lateral push toward the contralateral side were used in the present study.

### Measurement of infarct size

2,3,5-Triphenyltetrazolium chloride (TTC) staining was performed to determine the area of infarcted cerebral tissue caused by transient focal cerebral ischemia according to the method described in the previous study^29^. In brief, sections of the brain at 2 mm thickness were obtained from rats. Slices were immersed in 2% TTC/saline solution for 10 min at room temperature. Normal tissue appears red by TTC, whereas the infarct area is unstained, appearing white. The infarct area was analyzed by using Image J (NIH, Rockville, MD, USA).

### Primary cultures of rat cortical neurons

SD rats (embryonic day 16) were used for preparing primary cultures of cortical neurons according to Hayashi *et al*.^31^ with minor modifications. Briefly, cerebral cortices were removed into ice-cold Dulbecco’s-phosphate buffered saline (FUJIFILM Wako, Osaka, Japan). The tissue was minced and the cells were dissociated by incubation for 25 min at 37 °C in phosphate-buffered saline (PBS) containing 0.25% trypsin (Invitrogen). After trituration by using a fire-polished Pasteur pipet in Neurobasal medium (Invitrogen) containing 10% fetal bovine serum, the isolated cortical cells were then suspended in Neurobasal medium containing 0.5 mM glutamine, 2% B27 supplement (Invitrogen) and 1% penicillin-streptomycin (FUJIFILM Wako). These cells were plated at a density of 200,000 cells/well on 24-well plates (Falcon, Corning, NY, USA) pre-coated with poly-D-lysine (FUJIFILM Wako) and then cultured for 10 days prior to experiments. Cultures were maintained at 37 °C in a 5% CO_2_ incubator. One half of the medium was replaced with fresh neurobasal medium twice a week.

### Induction of cell injury by treatment with NMDA or glutamate

Cortical neurons in primary culture were washed twice with 250 µl/well Hank’s balanced salt solution (HBSS; Invitrogen) containing 2.4 mM CaCl_2_ and 20 mM HEPES without magnesium, which can block the NMDA receptor. The neurons were incubated for 15 min at 37 °C between each wash. Subsequently, the cells were incubated with the desired concentrations of NMDA or glutamate with 10 µM glycine, a co-activator of the NMDA receptor, in HBSS containing 2.4 mM CaCl_2_ and 20 mM HEPES without magnesium for 15 min at 37 °C. After treatment with NMDA or glutamate, the cortical neurons were cultured for the desired times in the culture medium. As the control experiments for NMDA or glutamate treatment, cortical neurons were incubated with HBSS buffer lacking either NMDA and glycine or glutamate and glycine. For the inhibition experiment, the cells were incubated with the NMDA receptor antagonist MK-801 for 15 min along with NMDA or glutamate at the same time. A potent calpain inhibitor, calpeptin was added 6 hours before the addition of NMDA. In the present study, age-matched cultured cortical cells without any treatment were used as the “untreated control group”.

### Assay of cell viability

Cell viability of cortical cells was determined by the XTT dye-reduction assay as previously described^32^ with minor modifications. The cells were incubated with 250 µg/ml XTT and 6.25 µM 1-methoy-5-methylphenazinium methyl sulfate in the culture medium for 1 h at 37 °C. Then, the culture media were transferred to a 96-well assay plate (Corning) for measurement. The absorbance at 450 nm was measured with a plate reader (EMax Plus Microplate Reader, Molecular Devices, San Jose, CA, USA). The relative cell viability was expressed as the ratio of the absorbance at 450 nm of each treatment group against that of the corresponding untreated control group.

### Immunoblotting

Rats were sacrificed by decapitation 24 h after surgery for preparation of cerebral ischemic rats. The ipsilateral and contralateral cortices were homogenized in ice-cold lysis buffer containing 320 mM sucrose, protease inhibitors, and a phosphatase inhibitor cocktail (Roche Diagnostics Co.) at 4 °C. Western blotting using total homogenates was performed according to standard protocols. Cultured cortical neurons were harvested in sample buffer containing 62.5 mM Tris-HCl, pH 6.8 10% glycerol, 2% SDS, and 5% β-mercaptoethanol. Western blotting using total homogenates and cytosolic fraction was performed according to standard protocols. Samples were heated at 95 °C for 5 min, and proteins were separated by SDS-PAGE and then transferred to polyvinylidene difluoride membranes at 80 V for 1.5 h. The membranes were incubated with 5% nonfat milk in 10 mM Tris-HCl, pH 7.4, containing 0.9% NaCl and 0.1% Tween 20 for 1 h at room temperature, and then incubated overnight at 4 °C with primary antibodies in 10 mM Tris, pH 7.4, containing 0.9% NaCl, 0.1% Tween 20, and 5% bovine serum albumin. Subsequently, the membranes were probed with horseradish peroxidase-conjugated secondary antibodies (dilution, 1:5000; Pierce Biotechnology, Rockford, IL, USA) for 1 h at room temperature. Immunoreactive proteins were detected by using ImmunoStar basic (FUJIFILM Wako), ImmunoStar zeta (FUJIFILM Wako) or SuperSignal^TM^ West Femto Maximum Sensitivity Substrate (34095, Pierce Biotechnology). The following primary antibodies were used: mouse anti-β-actin (a5441, Sigma), rabbit anti-LRP1 (dilution, 1:5000; 2703-1, Epitomics, Burlingame, CA), goat anti-LRP1 [C II] (dilution, 1:2000; AF2368, R&D systems, Minneapolis, MN, USA).

### Immunohistochemistry

On day 1 after surgery, the brain of cerebral ischemic rat was perfused with 4% paraformaldehyde in 0.1 M phosphate buffer via the heart. The brains were removed and immersed in 30% sucrose in 0.1 M phosphate buffer. Then they were cut into 4-mm-thick coronal slabs, which were subsequently embedded in OCT compound (4583, Sakurafinetek, Tokyo, Japan) and thereafter cut into 10-μm sections with a cryostat (CRYOSTAR NX50; Thermo Fisher). The sections were incubated overnight with the desired primary antibody at 4 °C after blocking, and then with the corresponding secondary antibody for 1 h at 25 °C. After a wash, the same section was incubated overnight with another primary antibody at 4 °C. Subsequently, it was incubated with the corresponding secondary antibody for 1 h at 25 °C. Omission of primary antibodies served as a negative control. No immunostaining was detected in this group. The following primary antibodies were used: rabbit anti-LRP1 (dilution, 1:5000; 2703-1, Epitomics) and mouse anti-TGN46 (dilution 1:200, ab2809, Abcam, Cambridge, UK) antibodies. The secondary antibodies used were as follow: Alexa Fluor 488-labeled donkey anti-rabbit IgG (Molecular Probes Inc., Eugene, OR, USA) and Alexa Fluor 594-labeled goat anti-mouse IgG antibodies (Molecular Probes Inc.). Fluorescence was detected by using an Olympus confocal fluorescence microscope (FV1000; Olympus). Fluorescent images were analyzed with the MetaMorph software program (Molecular Devices, Downingtown, PA). Based on background fluorescence and the size of their nucleus, anti-LRP1-labeled cells of the cerebral cortex were observed by use of the MetaMorph software program (5 sections per animal), which areas corresponded to coronal coordinates of 0.48 to 0.20 from bregma. The number of TGN46-positive cells, which were colocalized with ICD (β-chain of LRP1), were counted by use of the MetaMorph software program and the ratio of double-positive cells to Hoechst33342-stained cells was calculated.

### Immunocytochemistry

For immunocytochemistry, neurons (10 DIV) on glass coverslips pre-coated with poly-D-lysine were washed with PBS (300 μl) twice for 5 min each time and fixed with 4% paraformaldehyde at room temperature for 10 min. After having been washed twice with PBS (300 μl) for 5 min each time, the neurons were made permeable with 0.2% TritonX-100 in PBS (300 μl) by incubation at room temperature for 10 min and then blocked with a mixture of 10% normal goat serum, 1% BSA, and 0.2% Triton X-100 in PBS (300 μl) at room temperature for 1 h. The neurons were incubated with rabbit anti-LRP1 (dilution, 1:2000; 2703-1, Epitomics), goat anti-LRP1 [C II] (dilution, 1:100; AF2368, R&D), mouse anti-TGN46 (dilution 1:200, ab2809, Abcam) or mouse anti-furin (dilution 1:500; sc-133141, Santa cruz) in PBS containing a mixture of 10% normal goat or donkey serum, 1% BSA, and 0.2% Triton X-100 (300 μl) at room temperature for 1 h. The cells were washed 3 times with PBS (300 μl) for 5 min, and then incubated with Alexa Fluor 488-conjugated donkey anti-rabbit IgG (dilution 1:200, Thermo Fisher) and Alexa Fluor 594-conjugated goat anti-mouse IgG (dilution 1:200, Thermo Fisher), and donkey anti-goat IgG at room temperature for 1 h. Subsequently, the neurons were washed 3 times, 5 min each time, with 300 μl of PBS containing Hoechst33342 (0.5 μg/ml, 346-07951, Dojindo, Kumamoto, Japan) and mounted with Fluoromount/Plus (DiagnosticBioSystems, Pleasanton,CA). Fluorescence was detected by using a confocal fluorescence microscope (FV1000; Olympus). Four to ten images (435 × 330 μm) per experiment were randomly taken from 4 independent experiments and were analyzed with the MetaMorph software program (Molecular Devices, Downingtown, PA). The number of LRP1 (α-chain)-, TGN46- or furin-positive cells, which were colocalized with ICD (β-chain of LRP1), were counted by use of the MetaMorph software program and the ratio of double-positive cells to Hoechst33342-stained cells was calculated.

### Statistical analysis

All data were presented as the means ± standard deviation (SD) of the mean. Differences between 2 groups were evaluated statistically by use of the unpaired *t* test. Statistical analyses among multiple groups were performed using analysis of variance (ANOVA) followed by Tukey’s test as a *post hoc* test. P values of less than 0.05 were considered to indicate statistical significance.

## Supplementary information

Supplementary Information
