# Supplementary material for: Furin-mediated cleavage of LRP1 and increase in ICD of LRP1 after cerebral ischemia and after exposure of cultured neurons to NMDA
Source: Sci Rep. 2019 Aug 13;9:11782. doi: 10.1038/s41598-019-48279-x (PMC6692408; doi:10.1038/s41598-019-48279-x)
Supplement: Supplementary file 1 — Supplementary Information [file 41598_2019_48279_MOESM1_ESM.pdf]

Supplementary Information

Furin-mediated cleavage of LRP1 and increase in ICD of LRP1 after cerebral ischemia and after exposure of cultured neurons to NMDA

Mariko Yamada, Hideki Hayashi, Kaori Suzuki, Shoko Sato, Daisuke Inoue, Yui Iwatani, Meiko Ohata, Bo Yuan, and Norio Takagi

Department of Applied Biochemistry, Tokyo University of Pharmacy and Life Sciences, 1432-1 Horinouchi, Hachioji, Tokyo 192-0392, Japan.

Address correspondence and reprint requests to Dr. Norio Takagi, Department of Applied Biochemistry, Tokyo University of Pharmacy and Life Sciences, 1432-1 Horinouchi, Hachioji, Tokyo 192-0392, Japan. e-mail: takagino@toyaku.ac.jp

Supplementary Figure S1

Figure 1 (b)

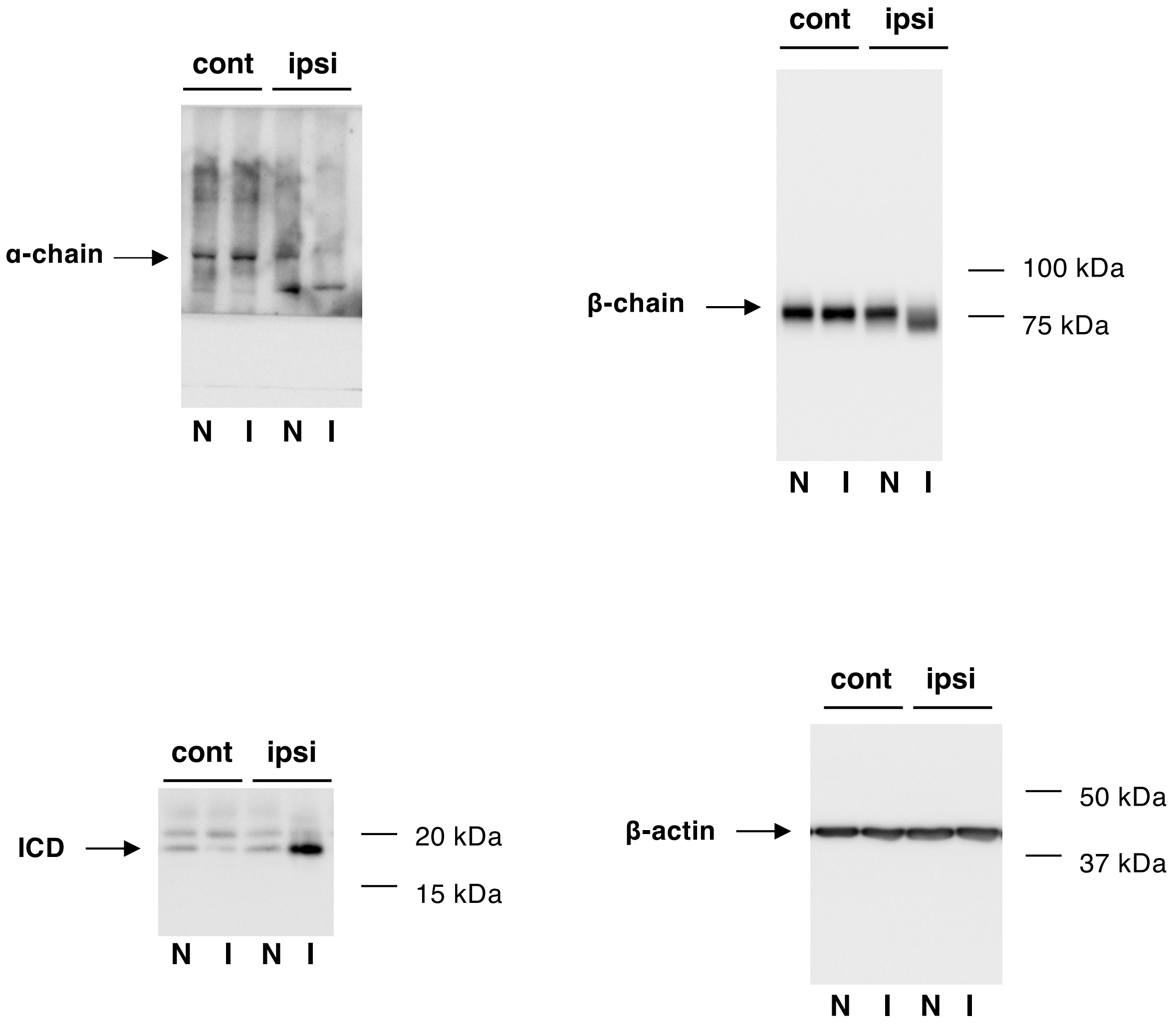

Supplementary Figure S1. Full-length blots corresponding to crop blots presented in the main manuscript

Full-length blots for Figure 1 (b) (α-chain, β-chain, ICD and actin) are shown.

Supplementary Figure S2

Figure 3 (a)

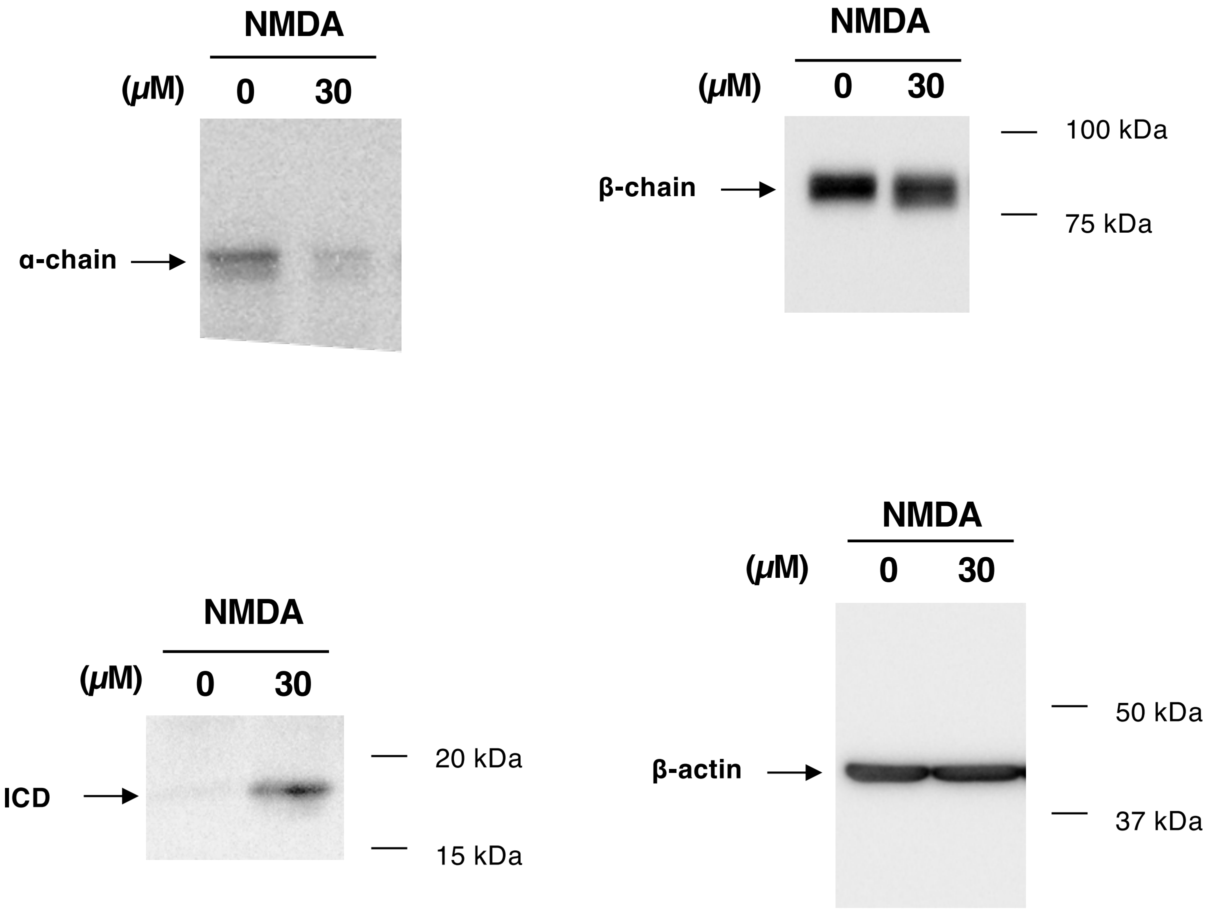

Supplementary Figure S2. Full-length blots corresponding to crop blots presented in the main manuscript

Full-length blots for Figure 3 (a) (α-chain, β-chain, ICD and actin) are shown.

Supplementary Figure S3

Figure 4 (a)

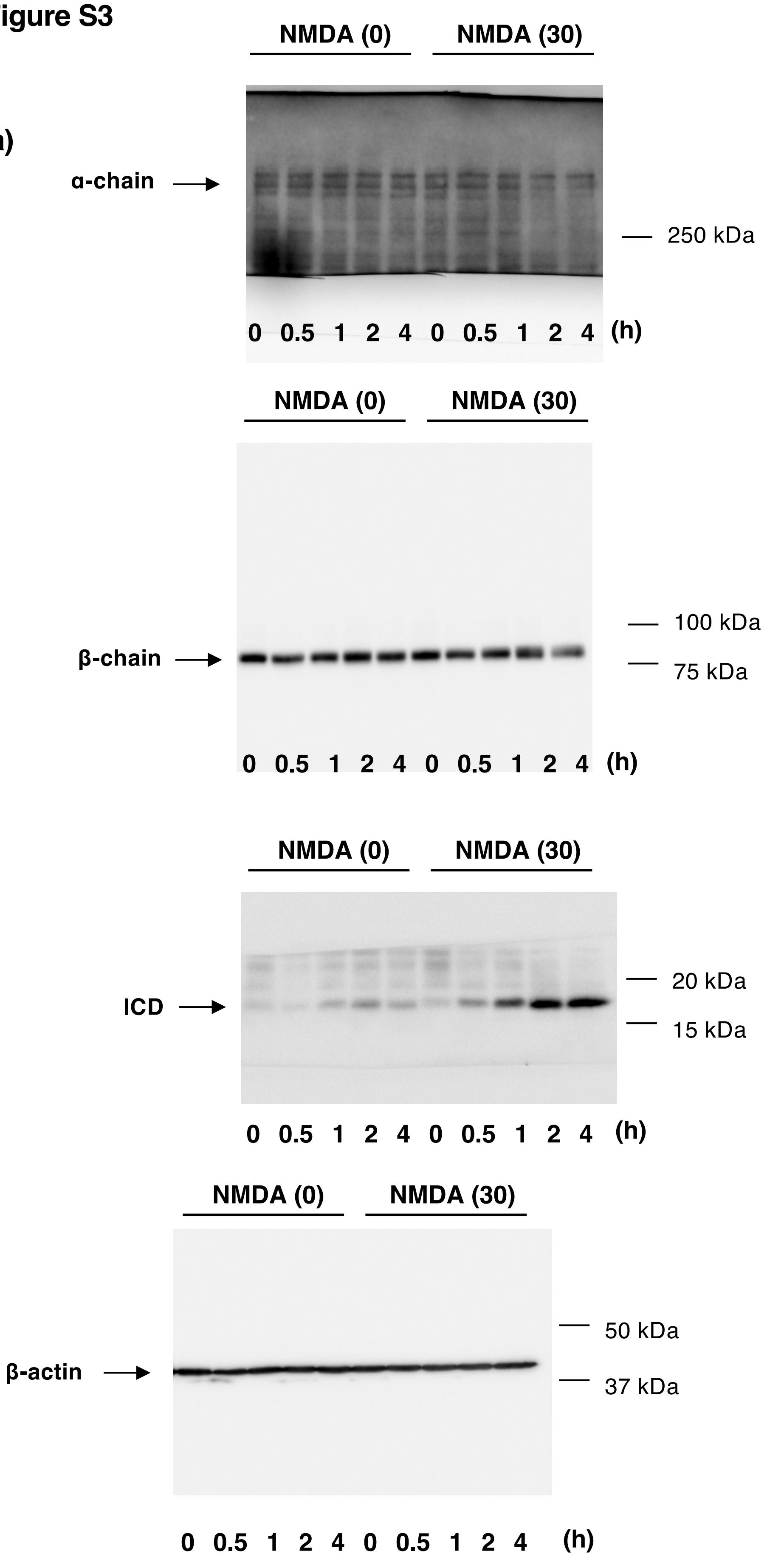

Supplementary Figure S3. Full-length blots corresponding to crop blots presented in the main manuscript

Full-length blots for Figure 4 (a) (α-chain, β-chain, ICD and actin) are shown.

Supplementary Figure S4

Figure 5 (a)

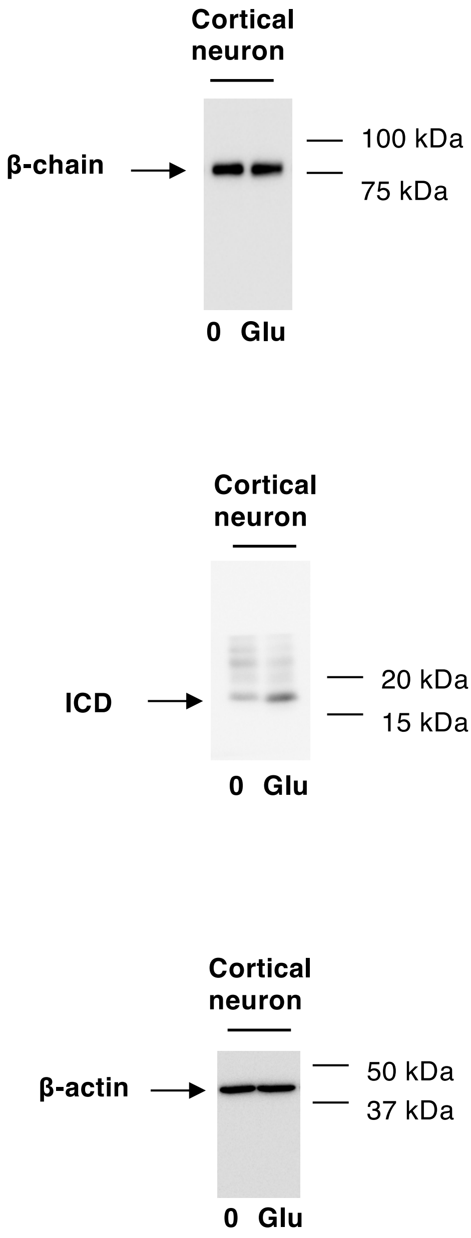

Supplementary Figure S4. Full-length blots corresponding to crop blots presented in the main manuscript

Full-length blots for Figure 5 (a) (β-chain, ICD and actin) are shown.

Supplementary Figure S5

Figure 6 (a)

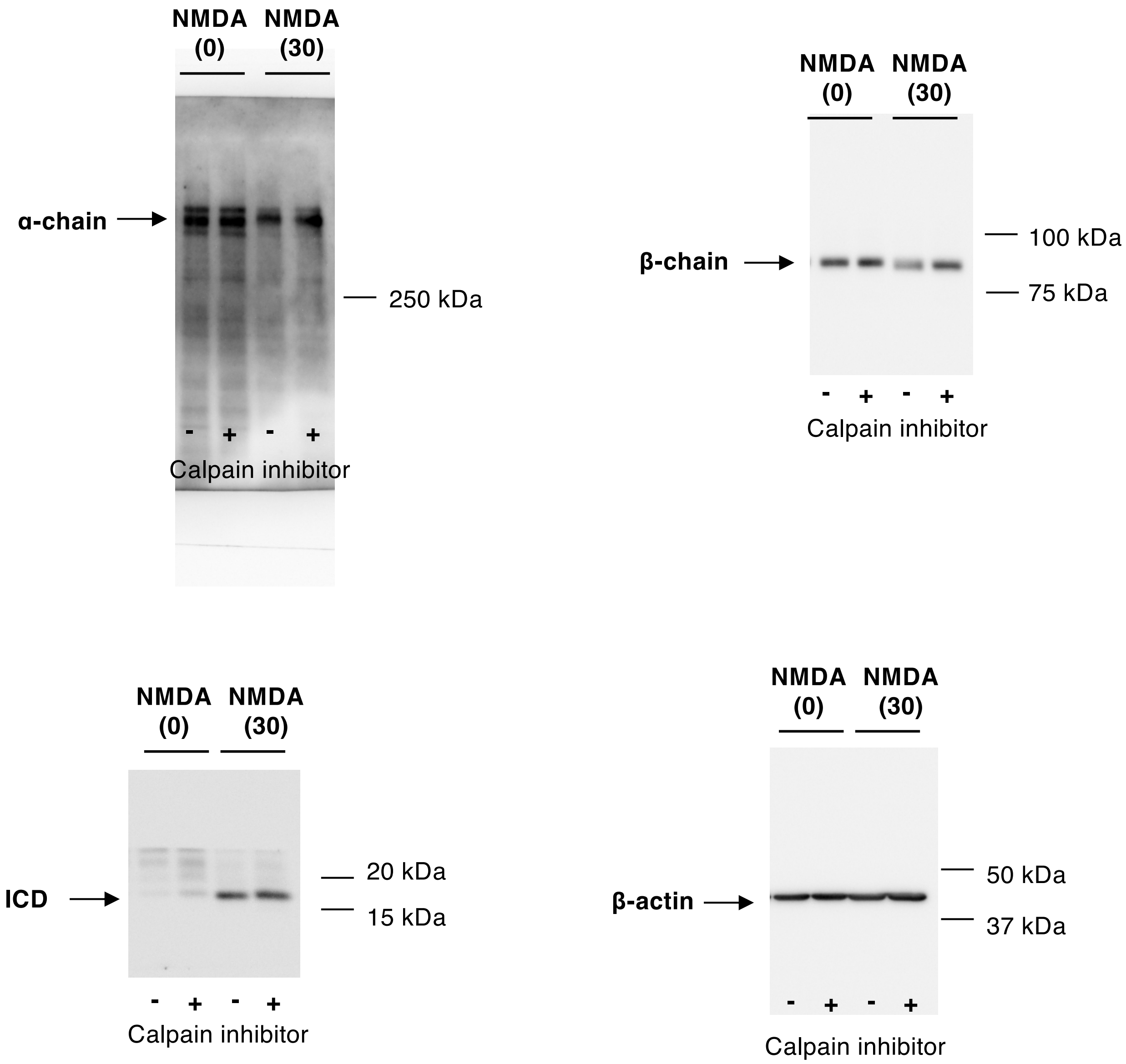

Supplementary Figure S5. Full-length blots corresponding to crop blots presented in the main manuscript

Full-length blots for Figure 6 (a) (α-chain, β-chain, ICD and actin) are shown.

Supplementary Figure S6

Figure 7 (a)

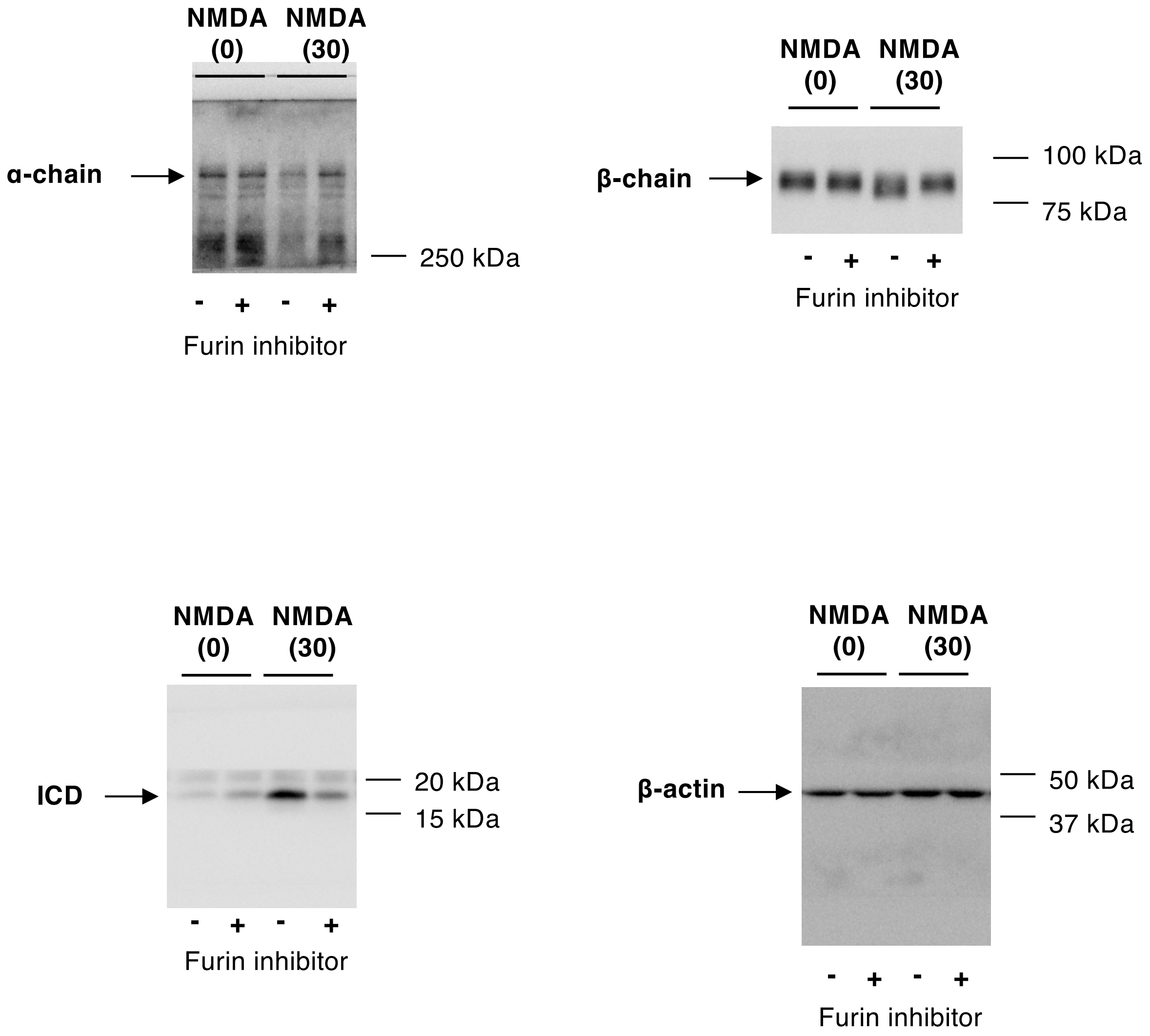

Supplementary Figure S6. Full-length blots corresponding to crop blots presented in the main manuscript

Full-length blots for Figure 7 (a) (α-chain, β-chain, ICD and actin) are shown.

Supplementary Figure S7

Figure 1 (f)

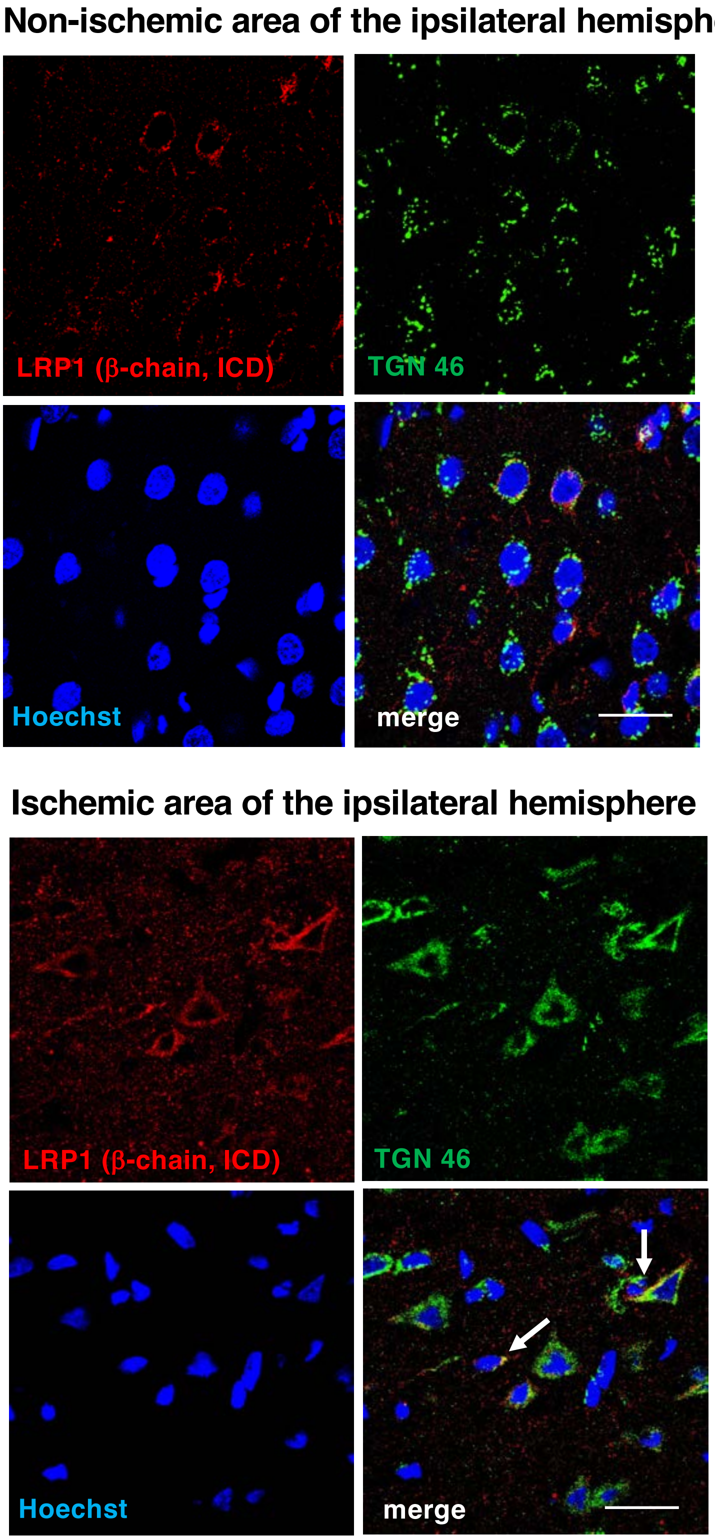

Supplementary Figure S7. Separate channels corresponding to merged photographs presented in the main manuscript

Separate channels corresponding to non-ischemic and ischemic area of the ipsilateral hemisphere for Figure 1 (f) (TGN 46, β-chain/ICD, Hoechst33342, and merged) are shown. The scale bar represents 30 µm.

Supplementary Figure S8

Figure 3 (e)

NMDA (0)

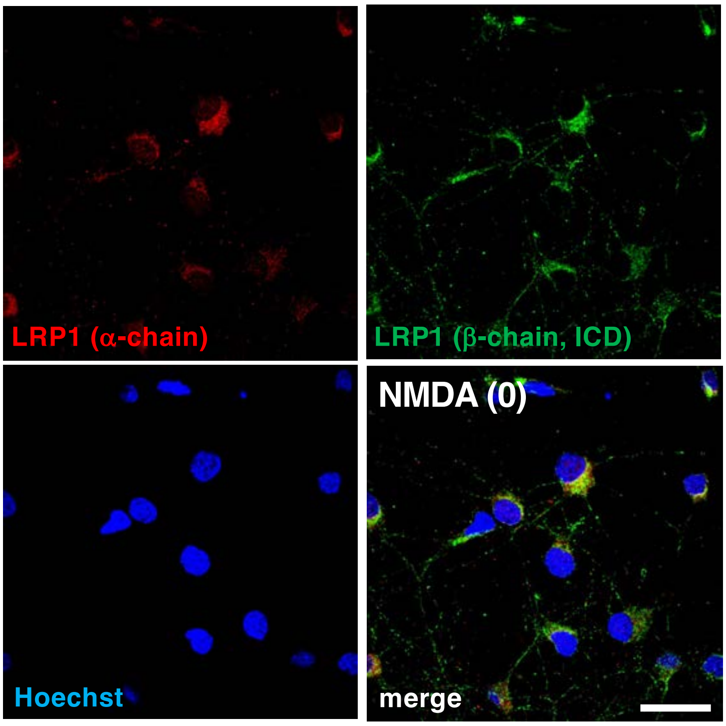

NMDA (30)

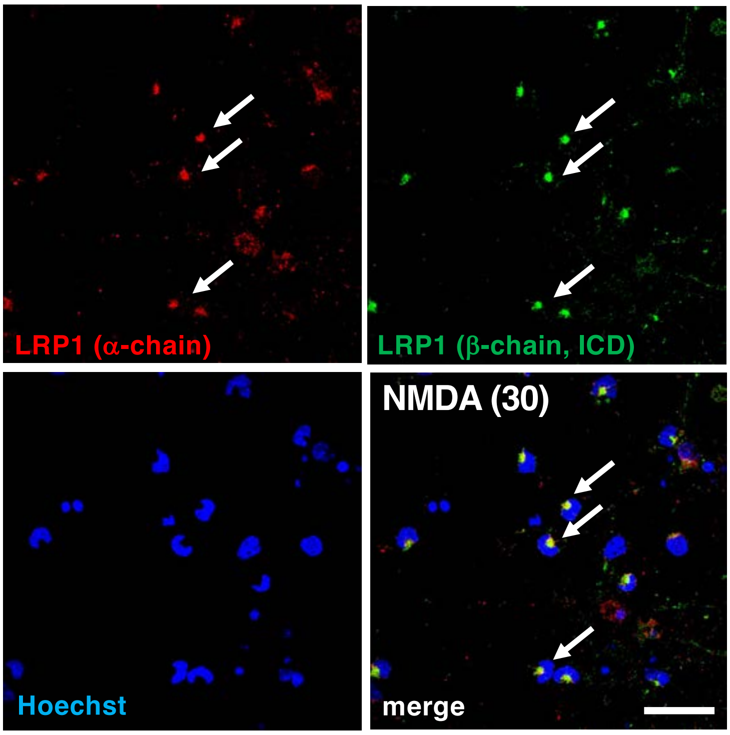

Supplementary Figure S8. Separate channels corresponding to merged photographs presented in the main manuscript

Separate channels corresponding to NMDA-treated (NMDA(30)) and -untreated (NMDA(0)) cortical neurons for Figure 8 (a) (α-chain, β-chain/ICD, Hoechst33342, and merged) are shown. The scale bar represents 30 μm.

Supplementary Figure S9

Figure 8 (a)

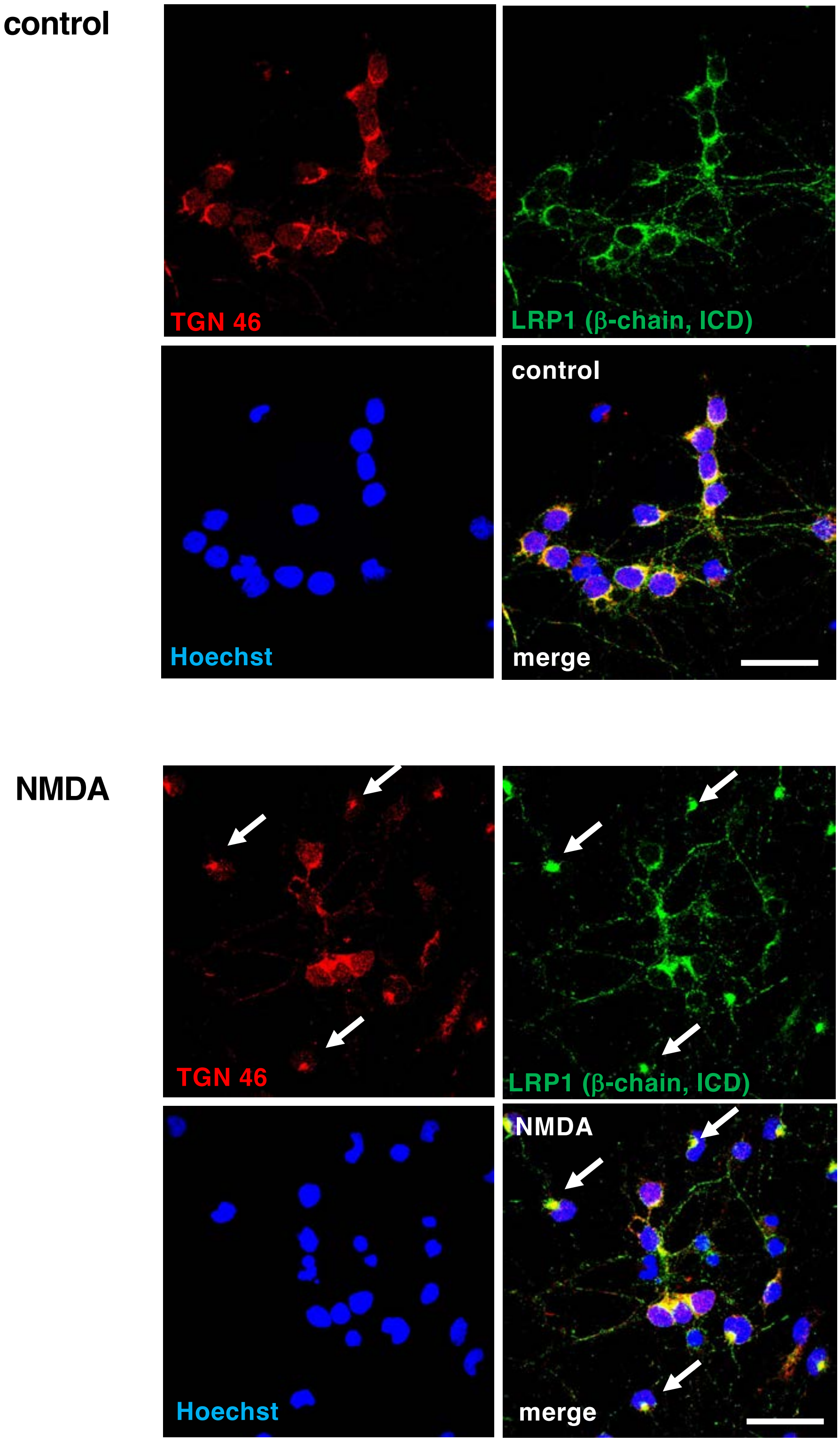

Supplementary Figure S9. Separate channels corresponding to merged photographs presented in the main manuscript

Separate channels corresponding to NMDA-treated (NMDA) and -untreated (control) cortical neurons for Figure 8 (a) (TGN 46, β-chain/ICD, Hoechst33342, and merged) are shown. The scale bar represents 30 µm.

Supplementary Figure S10

Figure 8 (c)

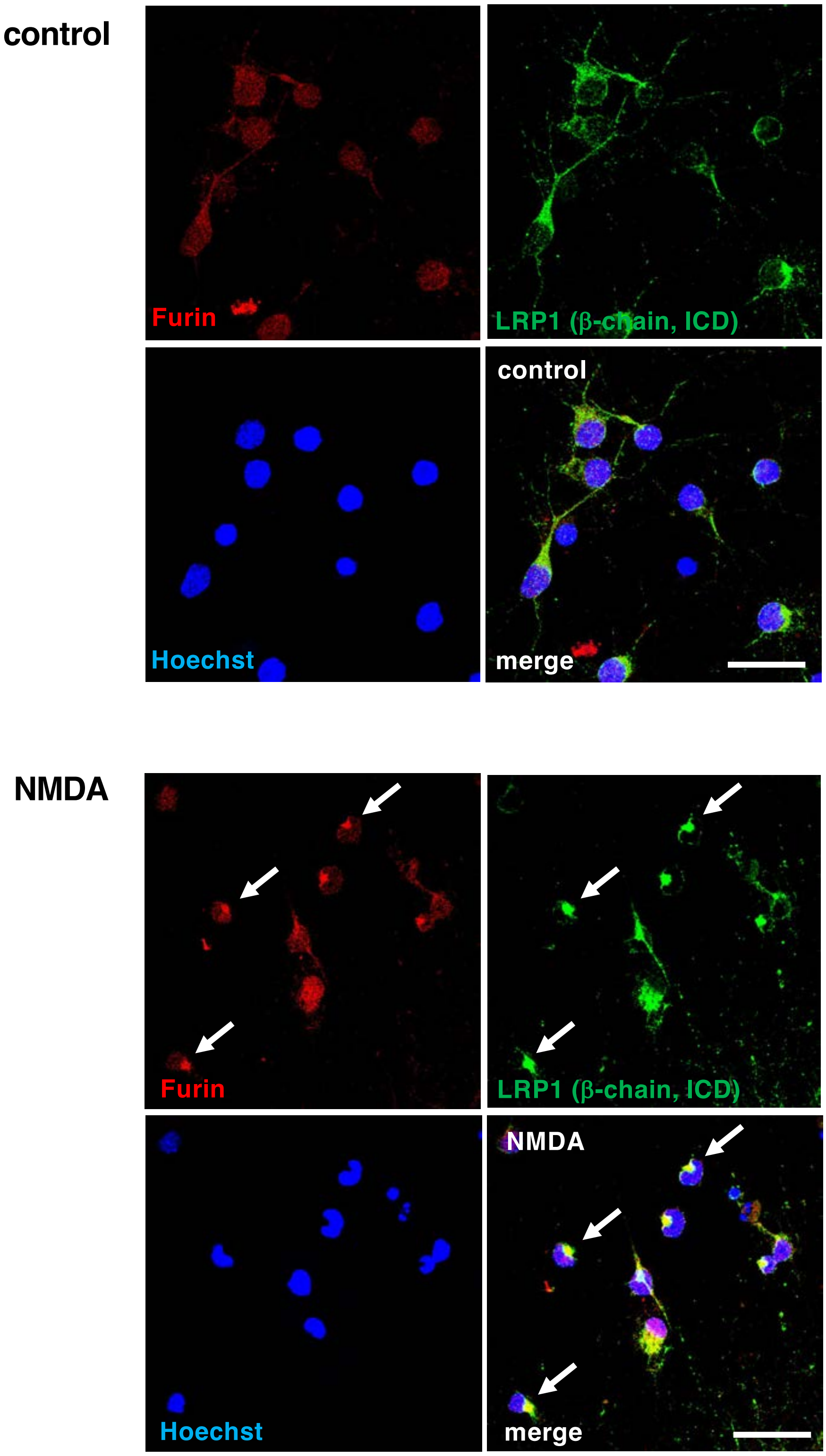

Supplementary Figure S10. Separate channels corresponding to merged photographs presented in the main manuscript

Separate channels corresponding to NMDA-treated (NMDA) and -untreated (control) cortical neurons for Figure 8 (c) (Furin, β-chain/ICD, Hoechst33342, and merged) are shown. The scale bar represents 30 µm.
